# Joint Relations Among Cannabis, Sleep, and Affective Symptomatology: A Scoping Review

**DOI:** 10.1007/s40429-026-00735-1

**Published:** 2026-04-16

**Authors:** Benjamin L. Berey, Sarah A. Thomas, Alexander W. Sokolovsky, Rachel L. Gunn, Mary Beth Miller

**Affiliations:** 1 Providence VA Medical Center, Providence, RI USA; 2 Center for Alcohol and Addiction Studies, Brown University School of Public Health, Providence, United States; 3 Department of Psychiatry and Human Behavior, Brown University Alpert Medical School, Providence, United States; 4 Bradley Hasbro Children’s Research Center, Providence, United States; 5 Carney Institute for Brain Science, Brown University, 164 Angell St., 4th Floor, Box 1901, Providence, RI 02912 USA; 6 Department of Psychiatry, University of Missouri, 1 Hospital Drive DC067.00, Columbia, MO 65212 USA

**Keywords:** Marijuana, Behavioral sleep medicine, Depression, Anxiety, Review

## Abstract

**Purpose of Review:**

This scoping review used a five-stage framework to evaluate empirical studies positing anxiety and depression as mechanisms or moderators in associations between cannabis use and sleep. Applicable peer-reviewed studies were identified in Google Scholar and PubMed using relevant search terms (e.g., cannabis, sleep, depression, anxiety).

**Recent Findings:**

Of the 20 articles included, most examined cannabis effects on sleep using prospective designs with adults recruited based on anxiety and/or depression symptomatology. Fewer studies tested bidirectional associations between cannabis and sleep. Among adults reporting clinically significant symptoms of anxiety and depression at baseline, certain cannabis product formulations were associated with concurrent and prospective improvements in subjective sleep phenotypes. However, this pattern of findings was not evident among those without anxiety/depression, none of the designs were experimental, and only one study included objective sleep measures.

**Summary:**

Providers should be aware that cannabis’ perceived sleep benefits are more pronounced among those with anxiety/depressive disorders. Experimental research with objective measures testing how and for whom sleep and cannabis are linked is needed.

## Introduction

 Cannabis’ therapeutic potential has been recognized globally for centuries and has been used in Western medicine since the 19th century [1, 2]. In North America, Canada’s 2018 Cannabis Act legalized cannabis nationwide, Mexico decriminalized recreational cannabis and legalized medical cannabis, and the United States (U.S.) remains in flux. California was the first state in the U.S. to legalize cannabis for medical purposes in 1996, and to date all but one state have at least partially recognized the medical benefits of cannabis [3]. Moreover, many European countries have enacted medical cannabis laws over the past decade [4]. Contemporaneously, epidemiologic data suggests that poor sleep health [5] has increased in recent decades [6, 7]. Further, sleep disorders such as insomnia are common across the lifespan with population estimates ranging from 6 to 30% depending on the sample, definition, and time-frame used [8–10]. See Table 1 for a glossary of relevant sleep terminology.

**Table 1:** Glossary of relevant sleep terminology

Insomnia	Sleep disorder characterized by difficulties falling asleep, staying asleep, and achieving good-quality sleep that impacts daytime functioning
Polysomnography (PSG)	Gold standard laboratory sleep assessment technique capturing brain activity, eye movement, and muscle tone
Rapid eye movement (REM) sleep	Sleep stage defined by REM, muscle paralysis, and brain activity comparable to wakefulness
Sleep architecture	Physiological sleep stages, including REM and non-REM sleep measured via polysomnography
Sleep efficiency (SE)	Percent of time in bed spent sleeping
Sleep health	Multifaceted pattern of sleep/wake behaviors across five dimensions (i.e., sleep duration, efficiency, timing, quality, and daytime functioning) that promote general well-being
Sleep latency (SoL)	Amount of time taken to fall asleep
Sleep quality	Subjective evaluation of good/poor sleep
Slow-wave sleep (SWS)	Stage of deep sleep indicated by low frequency and high amplitude waves
Total sleep time (TST)	Total amount of time spent asleep
Wake after sleep onset (WASO)	Amount of time spent awake after sleep onset and prior to final awakening

In the search for solutions to improve sleep, particularly those perceived as natural and safer than traditional pharmacotherapies, cannabis has emerged as a common sleep aid [11–16]. Despite no FDA-approved cannabis-derived drugs for its treatment, insomnia is among the myriad qualifying conditions for obtaining a medical cannabis card in many states [17–19]. For example, objective retail sales data from Colorado and Medicaid’s nationwide State Drug Utilization Data indicate that purchases and prescriptions of traditional sleep aids decreased following cannabis legalization (medical or recreational [20, 21]). Likewise, depending on the sample characteristics and time-frames used, survey data from diverse populations indicate that up to 85% of those who use cannabis do so for sleep, with up to 68% reporting daily use for sleep [11, 16, 22–24]. As such, a deeper understanding of how cannabis and sleep are related is necessary to develop more effective individual-, organizational-, and structural-level strategies to improve sleep health. Given individual differences in subjective evaluations of sleep quality, better understanding of cannabis effects on objective measures of sleep would be particularly helpful in informing healthcare decisions.

### Bidirectional Associations Between Cannabis and Sleep

**Fig. 1:**
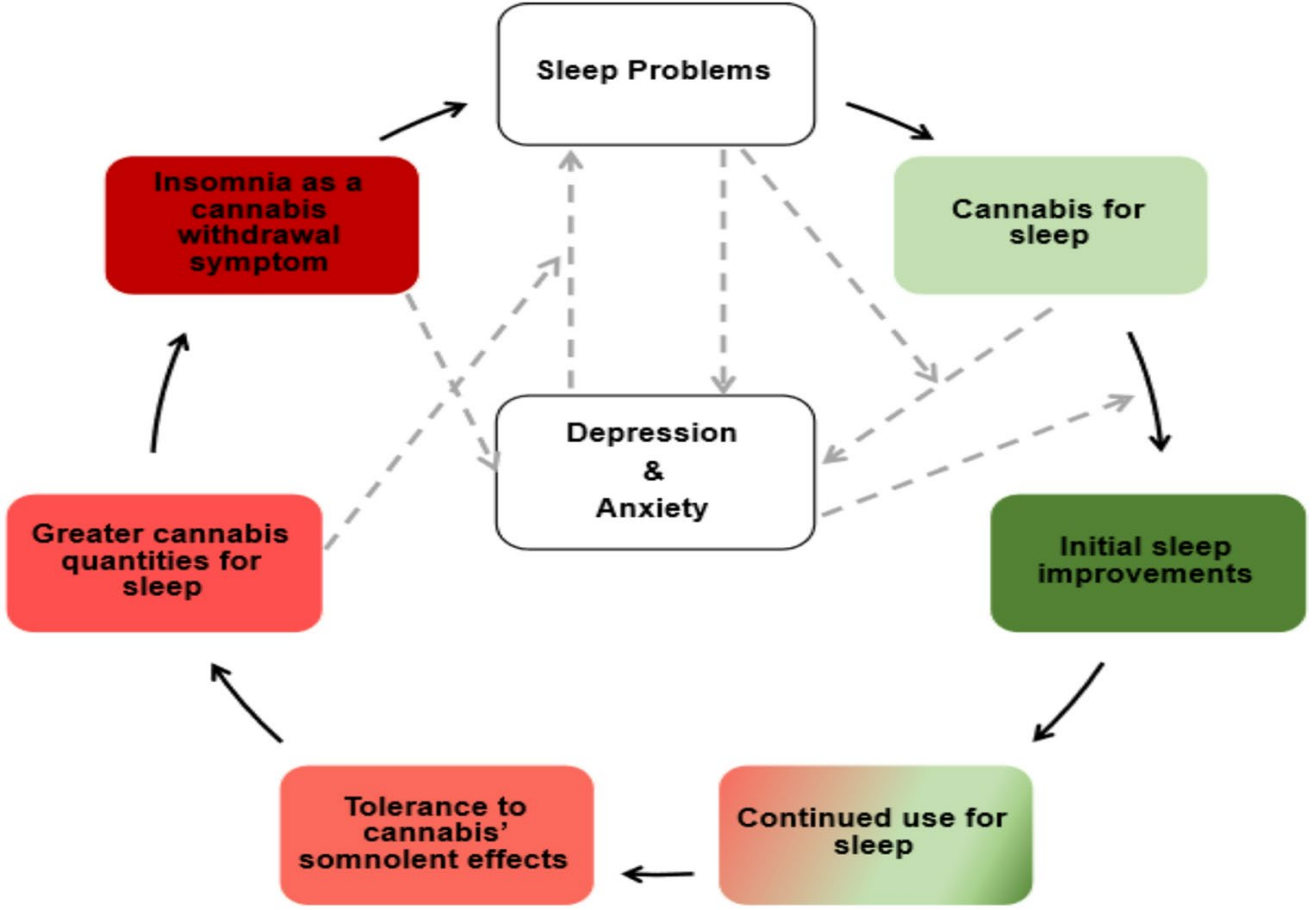
Theoretical model outlining reciprocal relations between cannabis use and sleep. Dashed arrows depict potential mediation and moderation pathways between cannabis, sleep, and depression and anxiety

Theory and empirical evidence highlight the bidirectional nature between cannabis and sleep [25–27] (see Fig. 1). That is, sleep disorders and poor sleep health confer risk for various cannabis-related outcomes, including, but not limited to, an earlier age of initiation, increased cannabis use frequency and related problems, cannabis use disorder (CUD) severity, and worse cannabis treatment outcomes [28–34]. Conversely, the two most commonly consumed cannabinoids (i.e., delta-9-tetrahydrocannabinol [THC; psychoactive] and cannabidiol [CBD; non-psychoactive]) may alter the sleep-wake cycle [35] in ways that improve certain sleep phenotypes in the short-term (e.g. [36, 37]), . Indeed, experimental and observational research measuring sleep via self-report or polysomnography (PSG) demonstrate that various cannabis formulations (e.g., whole-plant material smoked or vaporized, liquid THC administered orally) can reduce sleep onset latency (SoL) and wake after sleep onset (WASO), and increase sleep durations in both animals and humans [38–42].

While initial sleep improvements reinforce continued use, individuals can develop tolerance to cannabis’ sedative effects in as little as one week of daily use [43]. In turn, using cannabis in higher quantities impairs sleep architecture (e.g., poorer sleep efficiency [SE], longer REM latency, suppressed slow wave sleep [SWS]; [44–46]). Notably, cannabis’ sleep impairing effects are more pronounced in individuals with chronic use [31, 43, 47], or when used in larger amounts than usual among individuals who use less frequently (e.g. [42, 48]), . For example, adults reporting chronic, heavy cannabis use experience worse sleep efficiency, more sleep disturbances, and higher rates of insomnia measured via PSG and self-reports than adults with non-daily or no recent use [31, 47]. Finally, withdrawal-related sleep disturbances are common during periods of abstinence and can persist for several weeks, which in turn may motivate using cannabis for sleep again [49–52].

### Relevance of Affective Symptomatology to Links Between Cannabis and Sleep

There are many challenges to disentangling the nuanced associations between cannabis and sleep. First, sleep is a complex biological process consisting of behavioral and physiological phenotypes, the latter of which requires resource-intensive technologies (e.g., PSG) that are burdensome to participants. Second, cannabis products used in laboratory studies are limited by type, quality, and potency that are likely quite different from commercially available products with varying formulations and cannabinoid concentrations used for sleep [53–55]. Third, and perhaps most important, comorbid mental health conditions (e.g., anxiety, depression) are prevalent and potentially confounding variables in associations between sleep and cannabis use that make findings harder to interpret and generalize.

Interconnected theoretical and biopsychosocial frameworks indicate that understanding the multifaceted impact of depression and anxiety is key to elucidating some of the complex relations between cannabis use and sleep. First, it is critical to consider *why* individuals use cannabis, given the increased concomitant rates of depression, anxiety, and cannabis use in the US and worldwide [56–61]. Well-established motivational models of substance use and the self-medication hypothesis posit that cannabis use in the context of depression and/or anxiety symptoms is negatively reinforced by perceived improvements in sleep [62, 63]. Specifically, research investigating links between cannabis use, depression, and anxiety consistently indicates that coping motives (i.e., use for negative affect relief) reinforce continued substance use, with 30–60% of individuals reporting ‘medical’ use for depression or anxiety relief [54, 64]. Second, the hypothalamic-pituitary-adrenal (HPA) axis has been implicated in both stress and sleep [65], which could underlie sleep disruptions and internalizing disorders, leading to cannabis use for symptom relief. Similarly, recent research indicates a genetic link between sleep and cannabis use, such that a polygenic risk score for insomnia predicted an earlier cannabis use onset even after controlling for depressive symptoms [34]. As such, one can extrapolate that genetic risk for insomnia may also lead to cannabis use through the self-medication of internalizing symptoms with cannabis use.

### Cannabis and Depression/Anxiety

It is unsurprising that young adults with depression and anxiety use cannabis – perhaps to cope with negative affect – at considerably higher rates than their counterparts without such symptomatology [66, 67]. Although this evidence highlights the link from depression and anxiety to cannabis use, the reciprocal link is also well established. Epidemiological and cohort evidence suggests causal links between cannabis use and depression onset and symptom severity [68, 69], but less so for the onset of anxiety [70]. Taken together, evidence points to a strong reciprocal link between cannabis use with depression and anxiety. Critically, this relationship is closely intertwined with sleep.

Sleep disruption, including either hypersomnia or insomnia, is a canonical symptom in 60–90% of depressive episodes [71, 72]. Insomnia is frequently characterized by shorter sleep durations due to longer SoL and WASO^1^ [74, 75]. Similarly, 60–70% of individuals with generalized anxiety disorder (GAD) report sleep disruption, with increased SoL and WASO based on PSG studies [76]. Substantial evidence has accrued, however, that sleep disruption is not just an epiphenomenon of mood disorders, but can also precede their onset, including major depressive episodes and panic disorders as a prodromal precursor to both first- and recurrent-onset symptoms [71, 77]. In other words, cannabis use, sleep, and anxiety and depression likely exist in a complex intermediated web. For example, cannabis use may increase the risk for depression and anxiety by directly and/or indirectly disrupting sleep. While using cannabis to cope with negative affect may provide short-term sedative effects, tolerance requires more cannabis to experience the desired effects over time, which could lead to pharmacologically-mediated sleep disruptions. Alternatively, cannabis use could disrupt sleep, or worsen sleep disruptions, contributing to the development of anxiety and depression.

Biological frameworks support connections between sleep, cannabis use, anxiety and depression. For example, the endocannabinoid system (“ECS”) is responsible for prompting the release of wake-promoting neurotransmitters like dopamine that, at abnormal levels, may be associated with disrupted sleep, depression and anxiety [78–81]. Further, cannabis use during adolescent brain development – mediated by the ECS – is theorized to impact brain regions with high concentrations of ECS receptors and that play a role in sleep and the development of anxiety and depression (e.g., mesolimbic dopamine system, cerebellum; [82, 83]). Collectively, while direct links between cannabis use, sleep, depression, and anxiety have been investigated, there have been few attempts to synthesize this evidence into a holistic model.

Contemporary society’s interest in the therapeutic potential of cannabis for sleep necessitates a deeper understanding of how the two are related, including notable factors that may impact these relations. Recent reviews and meta-analyses have focused on cannabis and sleep generally [84–86], among specific populations (i.e., young adults [87]; cancer patients [88]), and as a treatment for sleep and anxiety disorders [89–92]. Yet, notable literature gaps remain, including how the presence of anxiety and depression impacts bidirectional associations between cannabis and sleep. As such, the overarching aims of this scoping review are to: (1) evaluate the recent empirical evidence on the interrelations between cannabis use, sleep, and anxiety and/or depression; (2) examine the different methodological approaches used in recent studies to investigate this topic, and; (3) identify future directions that can address salient knowledge gaps.

## Methods

The present scoping review followed an established five-stage framework [93] including: (1) Formulating the research question; (2) Identifying relevant studies; (3) Study selection; (4) Data charting, and; (5) Collating, Summarizing and Reporting the Results.

*Stage 1: Research Question.* This scoping review aimed to answer the following research question: What is the recent empirical evidence demonstrating how – or to what extent – depression and anxiety impact the bidirectional associations between cannabis and sleep? The present review chose to focus on studies examining THC because it is the most commonly used and commercially available cannabinoid. Likewise, findings focused on THC have the most relevant public health and policy implications due to its well-documented associations with adverse outcomes (e.g., CUD, psychiatric disorders [94, 95]). 

*Stage 2: Identifying studies*. Included studies focused on the intersection of cannabis, sleep, and depression or anxiety measured across various time periods (e.g., current, lifetime) and level of analysis (e.g., day- or person-level). We considered studies that measured: (1) cannabis across multiple domains (e.g., use frequency, quantity, problems, CUD symptoms, product preferences, and sleep-related use motives); (2) a broad range of sleep phenotypes including disturbances (e.g., sleep quality) and disorders (e.g., insomnia), dimensions of sleep health (e.g., sleep timing and duration), and chronobiology (e.g., circadian rhythms), and; (3) negative affective states that encompassed self-reports of depression and/or anxiety, major depression or anxiety disorder diagnoses, or varying levels of depression and/or anxiety symptom severity.

In Google Scholar and across the PubMed database, we searched peer-reviewed studies published from 2019 to 2024, written in English, that enrolled human participants. Relevant search terms included: “cannabis” OR “marijuana” OR “cannabinoid” OR “THC” AND “sleep” OR “insomnia” OR “sleep disorder” OR “polysomnography” OR “actigraphy” OR “circadian rhythm” OR “chronotype” AND “anxiety” OR “depression” with filters including “Species – Humans”. Reference lists from prior literature reviews and meta-analyses mentioned above were also hand-searched, resulting in 91 articles.

*Stage 3: Study Selection.* Study eligibility involved a two-step process determined by hand-screening the 91 articles identified in Stage 2. First, studies must have reported results from at least one statistical model testing associations between sleep and cannabis products with THC. Studies focused exclusively on sleep and cannabinoids other than THC (e.g., cannabidiol [CBD], cannabigerol [CBG]) were excluded. Second, studies were required to meet at least one of the following criteria: (a) enrolled participants based on depression and/or anxiety symptoms or diagnoses; (b) tested mediational pathways among cannabis, sleep, and anxiety or depression, or; (c) compared differences in sleep and/or cannabis use across groups based on depression/anxiety status, or varying levels of anxiety and/or depression within a single group. In total, 20 articles were included. Of the 71 excluded studies, *n* = 33 examined effects among cannabis, sleep, and depression/anxiety separately, *n* = 14 did not include a formal measure of depression and/or anxiety, *n* = 9 focused exclusively on cannabinoids other than THC (e.g. [96]), , *n* = 7 only reported on depression and/or descriptively, *n* = 5 sleep and/or cannabis use across groups based on depression/anxiety status, or varying levels of anxiety and/or depression within a single group, and *n* = 3 collected data on various reasons for using medical cannabis.

*Stage 4: Data Charting.* The authors reviewed all articles satisfying inclusion criteria and documented key study procedures, results, and other relevant information in a data charting form. Most studies recruited adults based on depression and/or anxiety symptoms or diagnoses to examine cannabis’ effect on self-reported sleep quality.

*Stage 5: Collating*,* Summarizing and Reporting the Results.* Primary results were collated to provide a narrative summary of the recent empirical literature focused on the intersection of cannabis, sleep, and anxiety and depression. Accordingly, results were grouped thematically by study sample (i.e., depression and/or anxiety as an eligibility criteria). Within each group, data were subsequently organized by study design (i.e., cross-sectional or prospective).

## Results

Twenty articles were included that reported on either the effects of cannabis on sleep among individuals with depression and/or anxiety symptoms or disorder diagnoses (*n* = 11), or moderation (*n* = 6) or mediational pathways (*n* = 3) among cannabis use, sleep, and depression and/or anxiety. Half of the studies (*n* = 10) included persons across the lifespan, whereas fewer studies included exclusively young adults (i.e., 18–30 years of age; *n* = 4) or middle age and older adults (i.e., 31 + years of age; *n* = 5), and only one study included adolescents younger than 18 years of age [97]. Only one study included both self-report and objective sleep measures. Of studies exclusively using self-report sleep measures, most (*n* = 12) used validated measures (e.g., Pittsburgh Sleep Quality Index [PSQI] [98]; Insomnia Severity Index [ISI; [99]), whereas six included one or more single-item questions. Most studies had prospective designs (*n* = 13), but few (*n* = 3) tested bidirectional associations between sleep and cannabis use. Table 2 includes detailed methodological information and sample descriptives for each study. Table 3 includes brief summaries of study results organized by sample, methodology, and direction of effects.

**Table 2:** Studies included in the present scoping review

Author (year)	Sample description (*n*)	Age range (in years)^a^	Sex (% Male)	Design	Sleep Variable(s)	Cannabis Measure(s)	Depression/Anxiety Measure(s)
Bapir et al. (2023) [113]	Adults with chronic pain only (*n* = 543) or with comorbid anxiety (*n* = 711)	Anxiety group: 44.8 (14.0) No anxiety group: 47.0 (14.9)	54.1	Prospective	Past-week sleep quality (SQS)	Use of CBMPs	GAD-7 EQ-5D-5L
Barbotin et al. (2023) [107]	Adults with a past-year major depressive episode (*n* = 2,864)	18+	not listed	Prospective	Lifetime sleep complaints (3 items assessing trouble falling asleep, early morning awakening, hypersomnia)	AUDADIS-IV CUD	AUDADIS-IV mood & anxiety disorders
Berey et al. (2024) [97]	Youth interested in reducing their cannabis use (*n* = 86)	19.80 (2.12), 15–24	41.2	Prospective	Self-reported prior-night trouble sleeping (single item)	Any use (y/n) Quantity (grams) Impaired control (y/n)	Self-reported negative affect (3 single-items)
Bidwell et al. (2024) [101]	Adults with anxiety (*n* = 347)	33.19 (13.24), 21–70	36.5	Prospective	Self-reported prior-night sleep quality (single-item)	Any use (y/n)	GAD-7 DASS-21
Coelho et al. (2023) [110]	College students (*n* = 14,787)	20.4 (2.3), 18–30	24.5	Cross-sectional	Self-reported past 3-month sleep complaints (4 single-items)	Past-year frequency (single-item)	Self-reported lifetime depression/anxiety diagnosis (single item)
Dokkedal-Silva et al. (2021) [111]	Adults living in Brazil (*n* = 793)	42.41 (14.33), 20–80	57.0	Cross-sectional	PSQI, ISI, PSG	ASSIST	BAI BDI
Ergisi et al. (2022) [103]	Adults with DSM-5 general anxiety disorder (*n* = 67)	37.42 (13.01)	67.2	Prospective	Past-week sleep quality (SQS)	Use of CBMPs	GAD-7 EQ-5D-5L
Kuhathasan et al. (2022) [108]	Adults with insomnia symptoms and depression (*n* = 100), anxiety (*n* = 463), or comorbid depression and anxiety (*n* = 114)	Depression: 30.93 (10.07), 18–62 Anxiety: 31.42 (8.91), 18–71 Comorbid: 33.98 (10.70), 18–62	44.5	Prospective	Self-reported insomnia symptoms	Self-reported product formulation and chemotype	Self-reported depression and/or anxiety
Livingston et al. (2023) [116]	College students with past-month cannabis use (*n* = 1,453)	19.61 (2.55)	29.8	Cross-sectional	Sleep motives (CMMQ)	Use frequency (MUG) CUDIT B-MACQ	DSM XC
Mangoo et al. (2023) [106]	Adults with a primary depression diagnosis (*n* = 129)	35.6 (11.1)	73.6	Prospective	Past-week sleep quality (SQS)	Use of CBMPs	GAD-7 EQ-5D-5L PHQ-9
Martin et al. (2021) [100]	Adults with depression or anxiety who used (*n* = 368) or did not use (*n* = 170) medicinal cannabis	46 (13)	20.6	Cross-sectional	PSQI global score	Self-reported product type, chemotype, dosing regimen, route of administration	HADS
Montebello et al. (2022) [114]	Adults with ICD-10 cannabis dependence (*n* = 128)	35.0 (10.9), 18–65	76.6	RCT	ISI total score	Past-month frequency (TLFB) Medication condition	DASS-21
Murphy et al. (2024) [104]	Adults with anxiety classified into impaired (*n* = 156) or not-impaired (*n* = 146) sleep cohorts	38.06 (11.7)	69.5	Prospective	Past-week sleep quality (SQS)	Use of CBMPs	GAD-7 EQ-5D-5L
Rifkin-Zybutz et al. (2023) [105]	Adults with a general anxiety disorder diagnosis (*n* = 302)	37.0 (11.5)	68.6	Prospective	Past-week sleep quality (SQS)	Use of CBMPs	GAD-7 EQ-5D-5L
Sznitman et al. (2024) [102]	Adults using cannabis for anxiety relief (*n* = 347)	33.15 (13.22), 21–70	36.3	Prospective	Self-reported prior-night sleep quality (single-item)	Any use (y/n)	DASS-21 Anxiety subscale
Tervo-Clemmens et al. (2023) [109]	Adults seeking cannabis for insomnia, pain, or anxiety / depressive symptoms (*n* = 181)	MCC Group: 38.26 (14.31), 18–65 WC Group: 36.69 (14.56), 18–65	34.4	Prospective	Self-reported prior-night sleep quality (single-item)	Any use (y/n) No. sessions	Self-reported depression (single item)
Walsh et al. (2024) [115]	Young adults from Southern California (*n* = 1926)	21, 20–23	39.1	Prospective	Past-month sleep problems (JSS)	Past-month and lifetime cannabis use frequency exposure	GAD-7 CES-D-10
Winiger et al. (2021) [34]	Adults with anxiety and lifetime cannabis use (*n* = 152)	31.45 (12.96), 21–70	31.7	Cross-sectional	PSQI	Past 14-day frequency (TLFB) Self-reported THC and CBD% (2 single-items) Cannabis-related sleep expectancies	DASS-21
Yau et al. (2019) [112]	Adults who used cannabis medicinally (*n* = 100)	18–30 (59%) 31–45 (21%) 46+ (20%)	68	Cross-sectional	PROMIS Sleep Disturbance Scale	Self-reported age of first use, conditions/ symptoms for medical use, preferred formulation and cannabinoid content, frequency, quantity, and time of day, negative effects	BDI PHQ-15 MINI
Yurasek et al. (2020) [117]	College students with past-month cannabis use (*n* = 267)	19.9 (1.4), 18–25	38.2	Cross-sectional	ISI total score	Past-month frequency (single-item) MCQ	DASS-21

*ASSIST* Alcohol, Smoking and Substance Involvement Screening Test, *AUDIT* Alcohol Use Disorder and Associated Disabilities Interview Schedule for DSM-IV, *BAI* Beck Anxiety Inventor, *BDI* Beck Depression Inventory, *B-MACQ* Brief-Marijuana Consequences Questionnaire, *CBD* Cannabidiol, *CBMP* Cannabis-Based Medicinal Product, *CES-D-10* Center for Epidemiologic Studies Depression Scale, *CMMQ* Comprehensive Marijuana Motives Questionnaire, *CUD* Cannabis Use Disorder, *CUDIT* Cannabis Use Disorder Identification Test, *DASS* Depression, Anxiety and Stress Scale, *DSM XC* DSM-5 Level 1 Cross-Cutting Symptom Measure–Adult, *DSM* Diagnostic and Statistical Manual of Mental Disorders, *EQ-5D-5 L* EuroQol-5 Dimensions-5 Levels, *GAD-7* Generalized Anxiety Disorder 7-item, *HADS* Hospital Anxiety and Depression Scale, *ICD-10* International Classification of Diseases, Tenth Revision, *ISI* Insomnia Severity Index, *JSS* Jenkins Sleep Score, *MCC* Medical Cannabis Card, *MCQ* Marijuana Consequences Questionnaire, *MINI* Mini-International Neuropsychiatric Interview, *MUG* Marijuana Use Grid, *PHQ* Patient Health Questionnaire, *PROMIS* Patient-Reported Outcomes Measurement Information System, *PSG* Polysomnography, *PSQI* Pittsburgh Sleep Quality Index, *SQS* Sleep Quality Scale, *THC* Δ^9^-tetrahydrocannabinol, *TLFB* Timeline Follow-Back, *WC* Waitlist Control

^a^sample(s) mean (sd), range (if provided), otherwise ns listed for each age category

**Table 3:** Bidirectional associations between cannabis and sleep, by sample and methodology

Samples recruited based on depression and/or anxiety symptoms or diagnosis	
Cannabis → Sleep	
*Cross-sectional*	*Prospective*
● Better self-reported sleep quality in adults who used medical CB vs. those who did not (Martin et al. 2021) [100]	● Use of CBMPs associated with better subjective sleep quality in adult patients with anxiety (Ergisi et al. 2022; Murphy et al. 2024; Rifkin-Zybutz et al. 2023) [103–105] or depressive (Mangoo et al. 2022) [106] disorders
● Current CB use and more frequent CB edible use associated with poorer self-reported sleep quality and efficiency, and shorter sleep duration (Winiger et al. 2021) [34]	● CB use days associated with better subjective sleep quality (Bidwell et al. 2024; Sznitman et al. 2024; Tervo-Clemmens et al. 2023) [101, 102, 109]
	● CB edible days associated with better subjective sleep quality than CB flower days; CBD-dominant CB products associated with better subjective sleep quality ratings than THC-dominant and balanced THC/CBD CB products (Bidwell et al. 2024) [101]
	● CB use improved self-reported insomnia symptoms (Kuhathasan et al. 2022) [108]
	● CB flower and oil products most effective for treating insomnia Sx in individuals with depression; All CB products equally effective for treating insomnia Sx in individuals with anxiety; all CB strains equally effective for treating insomnia Sx in individuals with depression or anxiety (Kuhathasan et al. 2022) [108]
Sleep → Cannabis	
*Cross-sectional*	*Prospective*
	● Hypersomnia increased odds of future CUD (Barbotin et al. 2022) [107]
	● Subjective sleep quality not associated with next-dayCB use (Bidwell et al. 2024; Sznitman et al. 2024) [101,102]

Samples not recruited based on depression and/or anxiety symptoms or diagnosis	
Cannabis → Sleep	
*Cross-sectional*	*Prospective*
● CB use increased odds of sleep problems overall; relations between CB use and specific sleep problems differed across YA with or without depression or anxiety (Coelho et al. 2023) [110]	● Greater CB use frequency associated with less sleep problems in YA with baseline depression or anxiety but more sleep problems in YA without baseline depression or anxiety (Walsh et al. 2024) [115]
● CB-related problems indirectly related to insomnia severity via negative mood (Yurasek et al. 2020) [117]	● Use of CBMPs associated with better subjective sleep quality in adult chronic pain patients with and without anxiety; CBMPs more effective for patients with moderate-severe anxiety vs. no anxiety (Bapir et al.2023) [113]
● Using medical CB to treat insomnia did not differ among adults w/ or w/o a lifetime anxiety or depression diagnosis (Yau et al. 2019) [112]	● CB use associated with higher ISI scores during and after Tx among persons with CUD (Montebello et al. 2022) [114]
Sleep → Cannabis (Cross-sectional)	Sleep → Cannabis (Prospective)
● Insomnia severity indirectly associated with CB-related problems via negative mood (Yurasek et al. 2020) [117]	● Youth with more trouble sleeping reported higher levels of negative affect, but nights with more trouble sleeping reduced odds of next-day CB use (Berey et al. 2024) [97]
● Subjective sleep quality and PSG-derived sleep architecture, or their interaction with depression/anxiety symptoms, not associated with risky CB use (Dokkedal-Silva et al. 2021) [111]	
● Depression and anxiety symptoms indirectly related to CB use frequency, problems, and CUD risk via cannabis-related sleep motives (Livingston et al. 2023) [116]	

*CB* Cannabis, *CBD* Cannabidiol, *CBMP* Cannabis-based medicinal product, *CUD* Cannabis usedisorder, *PSG* Polysomnography, *Sx* Symptoms, *Tx* Treatment, *YA* Young adults

### Evidence from Samples Recruited Based on Depression and/or Anxiety Symptoms or Diagnosis

Two cross-sectional studies tested effects of cannabis on sleep in adult samples with at least mild anxiety [34] or who had either depressive or anxiety disorders [100]. Nine prospective studies enrolled adults based on anxiety (*n* = 5; [101–105]) or depressive (*n* = 2; [106, 107]) symptoms or disorder, or both (*n* = 2 [108, 109]). All but one [107] study examined the effects of cannabis on sleep, with each providing evidence that cannabis improved various sleep phenotypes.

### Cross-Sectional Findings

#### Cannabis Use → Sleep

One study compared baseline PSQI-measured sleep quality and disturbances among those who did or did not report medical cannabis use among a large sample of primarily middle-aged adults with self-identified anxiety or depressive disorders [100]. Overall sample prevalence of anxiety (34%), depressive (15%), or both (51%) disorders did not differ across groups based on baseline cannabis use, but adults who used medical cannabis reported significantly lower past-month PSQI global scores (i.e., better sleep quality) than adults who did not use medical cannabis.

Another study examined associations between cannabis use and self-reported sleep outcomes among adults from the community with at least mild anxiety, lifetime cannabis use, and who wanted to use cannabis to treat anxiety [34]. More frequent use of cannabis edibles was associated with poorer sleep quality on the PSQI, controlling for age, alcohol use, current internalizing and stress symptoms, and gender. Current cannabis use and increased frequency of edibles was also associated with individual PSQI components: worse subjective sleep quality, worse subjective sleep efficiency, and shorter sleep duration. Yet, these associations did not remain significant when adjusting for the aforementioned covariates. With regard to cannabinoid profiles, THC concentration was not associated with PSQI scores, whereas greater CBD concentration was significantly associated with greater sleep efficiency and duration for older than younger adults, although similar to the other findings, the adjustments for covariates did not retain significance < .05.

Collectively, cross-sectional evidence linking cannabis to sleep was equivocal (e.g., better sleep quality [100] but more disturbances to specific sleep phenotypes [34]), although cannabis mode and cannabinoid profile may be relevant factors influencing these associations. Different study sample sociodemographic characteristics, measures used to quantify cannabis use, and depression and anxiety inclusion criteria likely also contributed to discrepant findings.

### Prospective Findings

#### Cannabis Use → Sleep

Several observational studies used secondary analysis from the United Kingdom Medical Cannabis Registry (UKMCR) to examine the direct effects of cannabis-based medical products (CBMP: i.e., prescribed medical flower or oil) on sleep. In three studies, middle-aged adults with a primary, secondary, or tertiary GAD diagnosis received CBMPs during an initial clinical assessment and reported on their past-week sleep quality at follow-ups up to one year later [103–105]. These studies consistently reported improvements in self-reported sleep quality, depression and/or anxiety at 1-, 3-, and 6- or 12-month follow-ups. Moreover, one of the studies examined whether cannabis’ efficacy varied based on participants’ baseline sleep quality (i.e., impaired vs. non-impaired based on an initial threshold score) [104]. Compared to adults without impaired sleep, adults with impaired sleep were significantly more likely to report clinically meaningful improvements in GAD symptoms at 1- and 3-month follow-ups and in sleep quality at all time points. Lastly, compared to adults without impaired sleep, adults with impaired sleep also exhibited a greater mean improvement in anxiety and depression symptoms. In yet another secondary analysis of UKMCR data in middle-aged adults with a lifetime depression diagnosis, CBMPs demonstrated improvements in self-reported sleep quality, depression, and anxiety at 1-, 3-, and 6-month follow-ups [106].

Additional prospective research in populations not receiving medical recommendations for cannabis supported its therapeutic potential for sleep. Two secondary analyses used data from an ongoing daily diary study where adults with mild to moderate anxiety self-selected a specific cannabis formulation (i.e., flower, edible) at baseline and were then randomized to a specific cannabinoid profile group (i.e., THC or CBD dominant, balanced THC/CBD) within their chosen formulation. Afterwards, participants completed daily surveys and were able to purchase and use their assigned cannabis product *ad libitum* from a local dispensary over one month. In the first study, participants reported better subjective sleep quality on cannabis use only days compared to alcohol only days, alcohol and cannabis co-use days, and non-use days [102]. Further, moderation analyses indicated that cannabis effects on day-level sleep quality were stronger for participants with more frequent cannabis use at baseline. However, main effects of cannabis formulation and cannabinoid profile group on subjective sleep quality were not statistically significant. In the second study, results indicated that cannabis use (vs. non-use) days were associated with better subjective sleep quality [101]. Further, the positive association between cannabis use and day-level sleep quality was stronger among those with higher baseline depression.

In another observational study, adults ages 18–71 with either depression, anxiety, or both used a medical tracking app to monitor insomnia symptoms before and after using cannabis [108]. In this study, the subsample with depression reported that cannabis effectively treated insomnia for most ages (i.e., with the exception of those over 45), that cannabis flower and oil were particularly effective relative to other products, and that all strains (i.e., CBD dominant, indica, sativa, and hybrid) were effective for self-reported insomnia. Results were even more all-encompassing for the subsample with anxiety, in that all strain and all products were perceived as effective. Finally, in the subsample with both anxiety and depression, there were beneficial effects for all age groups and for all strains and products. Overall, results suggest that individuals with both depression and anxiety report high efficacy of cannabis for self-reported symptoms of insomnia, but particularly among those with anxiety.

In a final daily diary study with adults ages 18–65 seeking cannabis for insomnia, pain, or anxiety/depression symptoms, participants were randomized to either obtain a medical card at the time of randomization or to a waitlist control condition (i.e., wait 12 weeks before obtaining card) [109]. Those who obtained a medical cannabis card at randomization showed improvements in sleep quality following randomization over time. Further, days where cannabis was used were associated with improved self-report sleep quality in the medical card group. No effects on depression were found.

Collectively, results provided consistent evidence that cannabis use is linked to improvements in self-reported sleep phenotypes acutely and over longer time periods at the between- and within-person level. However, data from randomized controlled trials that include placebo conditions are needed to further substantiate results from these observational studies. Importantly, cannabis formulation may have differential effects on sleep based on participants perceptions that cannabis edibles and CBD-dominant products optimally improved their subjective sleep quality.

#### Sleep → Cannabis Use

Results were equivocal among the three studies that prospectively examined effects of sleep on cannabis use [101, 102, 107]. In two daily diary studies, prior-night sleep quality was not associated with next-day cannabis use among adults with mild to moderate anxiety who were randomized to various cannabis products [101, 102]. Another study examined associations between sleep complaints (i.e., trouble falling asleep, early morning awakenings, and hypersomnia) and CUD in adults with a past-year major depressive episode using data from Waves 1 and 2 of the National Epidemiologic Survey on Alcohol and Related Conditions (NESARC) [107]. Baseline hypersomnia (i.e., Wave 1), but not trouble falling asleep or early morning awakening, increased odds of CUD three years later (i.e., Wave 2). Further, lifetime incidence rates for trouble falling asleep, early morning awakening, and hypersomnia were 67.6%, 43.3%, and 71.9%, respectively, among those who developed a CUD during the 3-year follow-up period.

Collectively, results provided mixed evidence for sleep disruptions as a predictor of future cannabis use. While sleep quality may not be related to cannabis use at the day-level, other sleep disruptions may increase risk for CUD over time.

### Evidence From Samples Not Recruited Based on Depression and/or Anxiety Symptoms or Diagnosis

Three cross-sectional [110–112] and three prospective [113–115] studies examined whether associations between cannabis and sleep were moderated by depressive and/or anxiety symptoms, whereas two cross-sectional [116, 117] and one prospective [97] studies tested mediational pathways linking cannabis, sleep, and depression and/or anxiety.

### Cross-Sectional Findings

#### Cannabis → Sleep

Three studies tested moderation or mediation pathways linking cannabis to sleep. First, in a large sample (*n* = 14,787) of French college students, greater past-year cannabis use frequency was associated with greater odds of past 3-month self-reported sleep problems (e.g., difficulty falling asleep/staying asleep; daytime sleepiness; poor subjective sleep quality, and insufficient sleep durations) regardless of prior depression or anxiety diagnosis [110]. However, among the subsample of participants with a history of depression or anxiety, those with daily cannabis use had higher odds of difficulty falling asleep/staying asleep, poor subjective sleep quality, and excessive daytime sleepiness than those reporting never/rarely using cannabis. In contrast, students *without* a history of depression or anxiety who reported daily cannabis use had higher odds of insufficient sleep durations than those reporting never/rarely using cannabis.

A second study recruited adults who used medical cannabis from a community dispensary to examine mental health and psychiatric symptom severity [112]. Participants most often reported using medical cannabis to treat anxiety (77%), insomnia (53%), and depression (47%). Exploratory analyses indicated that sleep disturbance ratings and the percentage of participants using medical cannabis to treat insomnia did not differ between those with or without a lifetime anxiety or depression diagnosis. Alternatively, participants with a lifetime anxiety diagnosis were more likely to report experiencing negative side-effects from cannabis than those without a lifetime anxiety diagnosis. The third study examined whether negative mood mediated the concurrent association between past-month cannabis-related problems and insomnia severity index (ISI) scores among undergraduate college students with current cannabis use [117]. Results indicated that cannabis-related problems were positively associated with negative mood, which in turn, was positively associated with insomnia severity.

Collectively, results provided mixed evidence for mediational and moderation pathways among cannabis use, depression and anxiety, and sleep. More frequent cannabis use may result in specific sleep disruptions for those with depression or anxiety, whereas depression and anxiety may be an underlying factor linking cannabis problems to insomnia severity for young adults.

#### Sleep → Cannabis Use

In one large-scale study, adults completed a self-report battery consisting of depression, anxiety, substance use, and various sleep measures followed by overnight PSG [111]. Structural equation models with subjective sleep quality (i.e., ISI, PSQI), sleep architecture (i.e., PSG-derived sleep staging, nighttime awakenings, total sleep time [TST], SOL, WASO), and psychiatric symptom (i.e., Beck Depression Index, Beck Anxiety Index) latent factors tested direct and interactive effects on risky cannabis use on the Alcohol, Smoking and Substance Involvement Screening Test (ASSIST; [118]). Neither main effects of sleep and psychiatric symptoms, nor their interaction, were associated with cannabis ASSIST scores.

In the aforementioned study with undergraduate college students, Yurasek and colleagues [117] also tested whether negative mood mediated the cross-sectional association between insomnia and past-month cannabis-related problems. Insomnia severity was positively associated with negative mood, which in turn, was positively associated with cannabis-related problems. Notably, negative mood’s indirect effect was stronger in males than females. A third study tested whether sleep motives mediated the cross-sectional association between affective symptoms and various cannabis use indices in young adult college students with current (i.e., past-month) cannabis use [116]. On average, the sample reported using cannabis for sleep almost half of the time. Further, depression and anxiety symptoms were positively associated with cannabis sleep motives, which in turn, was positively associated with cannabis use frequency, problems, and Cannabis Use Disorder Identification Test (CUDIT-R; [119]) scores.

Taken together, results from these studies provide evidence of the bidirectional nature between cannabis, *self-reported* sleep phenotypes, depression, and anxiety. For young adults, depression and anxiety may be an underlying factor linking insomnia severity to cannabis problems, or motivate the use of cannabis for sleep problems that imparts risk for negative cannabis outcomes.

### Prospective Findings

#### Cannabis → Sleep

In one secondary analysis from a prospective cohort study, young adults completed self-reports on past-month cannabis use, sleep problems, past two-week general anxiety symptoms, and past-week depressive symptoms at baseline and six months later [115]. Associations between cannabis and sleep problems differed based on participants’ baseline mental health status. Specifically, more frequent cannabis use was associated with fewer sleep problems at follow-up among participants meeting baseline clinical thresholds for anxiety or depression (i.e., GAD-7 and CES-D-10 scores > 10), but more sleep problems among participants without anxiety or depression. Further, more frequent cannabis use was associated with fewer six-month sleep problems when accounting for baseline sleep problems, but only among those who met clinical thresholds for anxiety or depression *and* reported sleep problems on 3 + days at baseline.

Another secondary analysis used UKMCR data from adults whose primary indication for CBMP treatment was chronic pain and who were classified into no anxiety and anxiety cohorts using baseline GAD-7 scores [113]. Improvements in subjective sleep quality from baseline were reported at 1-, 3-, and 6-month follow-up assessments regardless of anxiety cohort. However, patients with moderate or severe anxiety (i.e., GAD-7 scores *≥* 10) reported greater improvements from baseline in subjective sleep quality at 1- and 6-month follow-ups compared to patients in the no-anxiety cohort (i.e., GAD-7 scores < 5).

One randomized clinical trial (RCT) testing the effects of Nabiximols – a 1:1 THC: CBD extract – to reduce cannabis use in a sample with cannabis dependence examined secondary outcomes on sleep, depression, and anxiety [114]. While illicit cannabis use (i.e., use other than Nabiximols) during the trial and follow-up periods was associated with higher ISI scores, it did not significantly increase the odds of exceeding ISI clinical cut-off scores. Further, among those who had moderate-severe ratings of depression or anxiety and insomnia at baseline, a decrease in both symptoms occurred across the trial.

Collectively, study findings indicate that cannabis may improve subjective sleep quality among young adults or reduce sleep problems among primarily middle-aged adults with chronic pain, albeit with notable caveats. Further, cannabis use may exacerbate insomnia symptoms for individuals in cannabis use treatment.

#### Sleep → Cannabis Use

One secondary analysis tested mechanisms linking sleep disruptions to cannabis use during the pre-randomization period of a combined pharmacotherapy and psychosocial RCT in youth and young adults who were interested in reducing their cannabis use [97]. Participants completed daily morning reports of their prior day sleep and cannabis use, random reports measuring negative affect, and self-initiated reports each time they used cannabis for approximately one week. While youth with more trouble sleeping reported higher levels of negative affect, neither was associated with greater cannabis use during the pre-randomization period. Further, youth who reported more trouble sleeping the prior night had a lower the odds of using cannabis the next day.

## Discussion

Cannabis use for sleep has become increasingly prevalent in contemporary society, despite limited empirical evidence supporting its efficacy [19]. Our understanding of how bidirectional cannabis-sleep effects manifest in individuals with depression and anxiety is lacking. The present review evaluated the recent empirical evidence focused on cannabis use, sleep, and anxiety and/or depression. Additionally, we investigated methodological approaches used in these recent studies to identify future directions that can advance cannabis and sleep research.

### Key Points on the Role of Depression and Anxiety When Evaluating the Association Between Cannabis Use and Sleep

Overall, findings among individuals recruited based on depression and/or anxiety symptomatology consistently indicated that cannabis use is linked to improvements in subjective sleep quality and/or sleep disturbance cross-sectionally and longitudinally. Notably, there was some evidence to suggest that specific formulations and cannabinoid concentrations were perceived as more effective than others [101, 108]. Further, almost all reviewed studies contained adult samples who were, on average, between 30 and 40 years of age. Thus, while certain cannabis products may improve subjective sleep quality for middle-aged adults experiencing depressive or anxiety symptoms, more research is needed among individuals spanning different developmental periods (e.g., adolescence or older adulthood), given well documented differences in sleep patterns across the lifespan [120].

Alternatively, studies that did not recruit individuals based on depression and/or anxiety symptomatology produced largely equivocal findings. That is, cannabis use was associated with sleep problems and insomnia severity in some studies [110, 114, 117], improved subjective sleep quality and fewer sleep problems in other studies [113, 115], or was not associated with sleep altogether [112]. Key differences in sample sociodemographics and study methodologies likely contributed to inconsistent findings. For example, these studies implemented cross-sectional, prospective observational, and experimental designs with heterogenous samples spanning young adult college students [110, 115, 117] to middle-aged and older adults [113, 114], which make it difficult to draw conclusions about the complex associations between cannabis use, sleep, and depression and anxiety. Moreover, the majority of these studies did not use objective sleep methods.

Far fewer studies in this review examined sleep as a predictor of cannabis use. However, inconsistent with prior research (e.g. [28, 31, 32]), , results from studies included in the present review were largely equivocal regardless of whether individuals were enrolled based on depression and/or anxiety symptomatology. For example, the two studies linking sleep to cannabis-related problems and higher odds of future CUD focused on insomnia severity or self-reported hypersomnia [107, 117], whereas studies that combined self-reported and objective sleep measures [111] or measured day-level subjective sleep quality [101, 102] did not find significant associations with cannabis use indices. In yet another study, more trouble sleeping was associated with a lower odds of next-day cannabis use [97]. As such, more research to determine which sleep disruptions are most relevant to cannabis use and future negative outcomes is warranted.

### Clinical and Public Health Implications

When viewed collectively, study findings can inform future clinical practice and public health initiatives by providing insights about potential mechanisms linking cannabis to sleep and how depression and anxiety can moderate these associations. Specifically, elevated depression, anxiety, and stress may be more relevant to explaining why cannabis-related problems confer risk for sleep disorders like insomnia in young adult college students [117] but less so for how sleep disruptions relate to cannabis use among adolescents. Likewise, adults who are physically dependent on cannabis and reduce their use may also experience reductions in depression, anxiety, and insomnia [114]. Notably, findings from this review underscore the considerable nuance for how cannabis may impact sleep in individuals with anxiety or depression. For example, while cannabis may improve subjective sleep quality for individuals with higher levels of anxiety, daily use may actually exacerbate sleep disruptions [110] or be less effective in individuals who report good sleep at baseline [115]. Thus, clinicians should carefully consider a patient’s current severity of sleep disruptions – or lack thereof – when discussing cannabis use in the context of anxiety or depression and explain how cannabis may have diminishing returns as a sleep aid when used too frequently.

### Recommendations to Advance Cannabis and Sleep Research

Several key future research areas emerged after examining the methodologies used by the studies included in the present review. First, all but one study exclusively measured self-reported sleep outcomes – often subjective sleep quality – and many studies contained samples who were seeking cannabis use for sleep. Indeed, in many of the prospective studies included in this review, participants were either prescribed a CBMP or were randomized to a condition where they could purchase and use cannabis from a licensed dispensary for insomnia, depression, and/or anxiety. Given near universal perceptions that cannabis is an effective sleep aid [12, 16, 121] coupled with the fact that in many studies participants were prescribed cannabis explicitly for their sleep (e.g. [103, 113]), , expectancy effects (i.e., expectations for improved sleep in line with self-reported improvements) were likely to be strong in these samples. Thus, to adequately account for sleep-related cannabis expectancies, future research should use experimental placebo-controlled designs to separate effects (i.e., cannabis from expectancy) on sleep. Second, given the multifaceted nature of sleep, use of valid and reliable subjective and objective assessments are critical – and recommended [122] – in mechanistic research studies. The importance of including both subjective and objective measures was also emphasized in two recent reviews on sleep and substance use disorders [123, 124].

Third, many of the studies included in this review contained samples with wide age ranges that may have confounded results given well-documented age-related changes in sleep patterns [120], and only one study enrolled adolescents [97]. Thus, more research is needed to ascertain relations among cannabis, sleep, depression and anxiety across unique developmental periods generally, and during adolescence specifically. For example, future research should carefully consider age-related eligibility criteria or implement study designs stratifying participants based on developmental stage. Fourth, while this review evaluated recent empirical literature focused on how depression and anxiety impact links between cannabis and sleep, there is clinical and theoretical value in examining how cannabis and sleep impact anxiety and depression. For example, in addition to improved subjective sleep quality, several studies using UKMCR data found that CBMPs concomitantly reduced depression and anxiety symptoms over time [103–106, 113]. Yet, it is unclear whether better subjective sleep quality led to fewer depression and anxiety symptoms or if the effects of CBPMs on these outcomes operated independently.

Lastly, most studies in the present review measured cannabis use frequency/quantity or severity. Given psychometric concerns of existing cannabis quantity measures (e.g. [125]), , and the emergence of myriad novel cannabis products with unique formulations and cannabinoid compositions, a critical next step is to examine how these factors impact sleep among individuals with varying levels of depression and anxiety. To this end, future research would benefit from measuring cannabis quantity based on standardized THC units that can be applied across all cannabis products [126]. Indeed, more detailed assessments will provide added granularity given that the duration and intensity of subjective effects differ across both administration mode and dose [127, 128].

## Conclusion

As a growing cannabis and sleep literature emerges, recent work demonstrates the importance of considering how depression and anxiety can impact the bidirectional associations between cannabis and sleep. In summary, based on recent research findings there appears to be positive association between cannabis use and sleep quality, particularly among middle-aged adults with anxiety and depression. Yet, more research utilizing experimental designs with adequate control conditions is needed, particularly in adolescent populations. Further, ascertaining mechanisms linking the ECS to sleep behavior and physiology and the pharmacological effects of distinct cannabis modes and formulations on sleep remains an important research endeavor.

## Key References

- Dokkedal-Silva V, Fernandes GL, Morelhao PK, Pires GN, Rowlett JK, Galduroz JCF, et al. Sleep, psychiatric and socioeconomic factors associated with substance use in a large population sample: A cross-sectional study. Pharmacology Biochemistry and Behavior. 2021;210:173274. This study used self-reported and objective sleep phenotypes to comprehensively examine relations among cannabis, sleep, and depression and anxiety.
- Kuhathasan N, Minuzzi L, MacKillop J, Frey BN. An investigation of cannabis use for insomnia in depression and anxiety in a naturalistic sample. BMC psychiatry. 2022;22(1):303. This study used patient-reported outcome data to demonstrate how different cannabis products impact self-reported insomnia symptoms.
- Walsh CA, Euler E, Do LA, Zheng A, Eckel SP, Harlow BL, et al. Cannabis use and sleep problems among young adults by mental health status: A prospective cohort study. Addiction. 2024. This study demonstrates that links between cannabis and sleep problems differ based on baseline mental health status.

## Data Availability

No datasets were generated or analysed during the current study.

## Footnotes

1 Hypersomnia symptoms were present in up to 50% of individuals with a past-year Major Depressive Episode, and up to 30% of individuals experienced both hypersomnia and insomnia [73]. Although factors related to hypersomnia are also of key scientific interest, we focus here on insomnia symptoms which are both more prevalent and serve as a common motive for medical cannabis use.
